# Acquired partial lower urinary tract obstruction caused by intravesical ureterocele in an adult dog

**DOI:** 10.1111/jsap.13489

**Published:** 2022-02-24

**Authors:** A. Uva, F. Gernone, M. A. Cavalera, P. D'Ippolito, M. Ricciardi, G. Carelli, A. Zatelli

**Affiliations:** ^1^ Department of Veterinary Medicine University of Bari “Aldo Moro” Strada Provinciale per Casamassima km. 3 70010 Valenzano Italy; ^2^ Laboratorio ACV Triggiano Via C. Battisti 255/257 70019 Triggiano Italy; ^3^ Private Practitioner CT‐MRI Support Service Via dei Glicini 11 70016 Noicattaro Italy; ^4^ Centro Veterinario Polidiagnostico Via A. Stradella 49 76123 Andria Italy; ^5^ Ospedale Veterinario S. Fara Via Generale Nicola Bellomo 91 bis 70124 Bari Italy

## Abstract

Ureterocele is an uncommon condition in dogs characterised by a cystic dilation of the submucosal portion of the distal ureter. A 4‐year‐old intact male Siberian husky with a 4‐month previous diagnosis of ureterocele was presented for pollakiuria. Abdominal ultrasound showed increased dimensions of the ureterocele, and a retrograde positive contrast urethrocystography detected a filling defect of the bladder neck lumen. The position of ureterocele was considered responsible for the partial urinary obstruction. This hypothesis is supported by the resolution of pollakiuria after surgical ureterocele resection. Based on a literature search, this is the first case of an intravesical ureterocele causing partial urinary obstruction in dogs. Ureterocele should be considered as a differential diagnosis in patients with pollakiuria.

## INTRODUCTION

Ureterocele is defined as a cystic dilation of the submucosal portion of the distal ureter (McLoughlin *et al*. 1989), involving one or both ureters, and thus defined as unilateral or bilateral (McLoughlin *et al*. 1989). From an aetiological point of view, the cause is still unknown although several theories have been proposed to explain its congenital origin (Stephens 1971, Tanagho 1976). Ureteroceles typically cause at least partial ureterovesicular obstruction resulting in secondary obstructive nephropathy and progressive loss of renal function (Timberlake & Corbett 2015). Moreover, recurrent urinary tract infections (UTIs) causing pyelonephritis can result in damage to the renal parenchyma, loss of kidney function and chronic kidney disease (Sutherland‐Smith *et al*. 2004). The most clinical signs most commonly related to ureteroceles are secondary to obstructive nephropathy, associated ureteral ectopia and UTIs, including dysuria, haematuria and urinary incontinence (Sutherland‐Smith *et al*. 2004). Once identified, the ureterocele should be treated to prevent complications.

In human medicine, the American Academy of Pediatrics classified ureteroceles as *intravesical*, if the cystic dilation is entirely located within the bladder, or *ectopic* if it is partially and permanently situated in the bladder neck or in the urethra (Glassberg *et al*. 1984). An ectopic ureterocele grading system based on the degree of renal parenchymal jeopardy (*i.e*. renal units at risk of damage related to obstruction) was suggested by Churchill *et al*. (1992). In veterinary medicine, Stiffler *et al*. (2002) proposed an anatomical and functional classification system for dogs in accordance with Glassberg *et al*. (1984) and Churchill *et al*. (1992), respectively.

Although confirming the anatomical *intravesical* classification of the ureterocele, Stiffler *et al*. (2002) defined as *ectopic* an ureterocele located in an abnormal position and associated with an ectopic ureter, thus considering for the first time the location of the corresponding ureteral outlet. On the other hand, from a functional point of view, Stiffler *et al*. (2002) adapted the classification proposed by Churchill *et al*. (1992) to dogs, staging ureteroceles based on renal injury associated with reflux or urine flow obstruction. This modified functional classification of ureteroceles considered the patient's prognosis (Stiffler *et al*. 2002) but did not take into consideration possible lower urinary tract diseases (LUTDs).

Published research outputs on the description of ureterocele in dogs were collected from PubMed (http://www.ncbi.nlm.nih.gov/pubmed/) and Web of Science (http://www.webofscience.com) databases on January 11, 2022. The key words used were “ureterocele,” “dog” and “canine.” The search of PubMed was conducted using the Boolean operators AND and OR [ureterocele AND (dog OR canine)]. Thirty‐one original papers published from 1971 to 2021 were returned from PubMed and Web of Science databases. In addition, the book by Ettinger *et al*. (2017) was searched.

No cases of lower urinary tract obstruction caused by intravesical ureteroceles in dogs were found during these searches.

## CASE HISTORY

**FIG 1:**
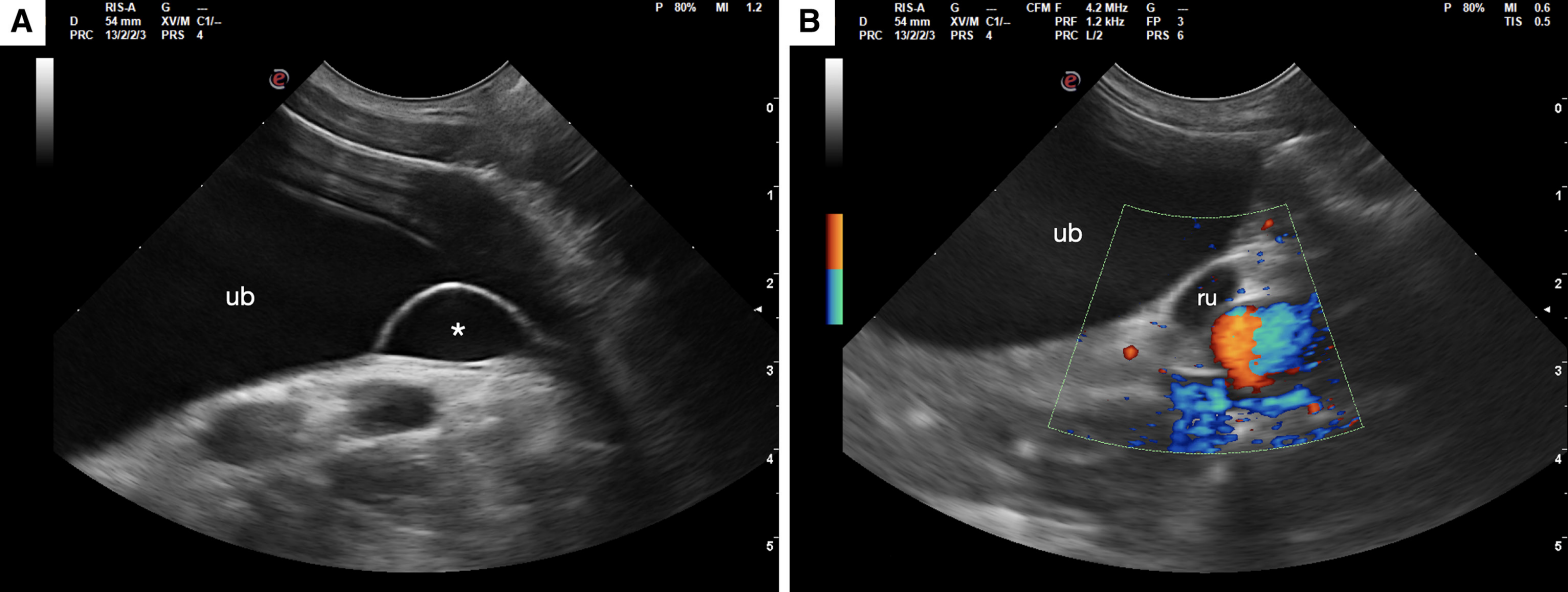
Sagittal ultrasonographic image of the urinary bladder (ub). (A) Smooth fluid‐filled lesion (approximately 2.2×0.5 cm) protruding into the bladder close to the trigone region is visible, consistent with a ureterocele (*). (B) The distal portion of the right ureter (ru) shows a mild dilation of 4.6 mm

A 4‐year‐old Siberian husky, intact male, 32 kg bodyweight, was referred because of a 2‐year history of intermittent gross haematuria and struvite crystals at urinalysis. The physical examination, complete blood count (CBC) and complete biochemical panel were normal. Urinalysis performed on a urine sample obtained by cystocentesis revealed more than 50 RBC/hpf at urine sediment examination, together with a negative urine bacterial culture. Abdominal ultrasound (US) identified a thin‐walled cystic lesion (approximately 2.2×0.5 cm) protruding into the urinary bladder close to the trigone region (Fig 1A). A mild right pyelectasis and severe ipsilateral hydroureter were also identified (Fig 1B). Based on the anatomical location, shape of the cyst, right pyelectasis and ipsilateral hydroureter, a grade 2 (Stiffler *et al*. 2002) right ureterocele was considered as the most likely differential diagnosis.

As the US did not clearly visualise the ureteral jets and in order to better define the potential relationship between the cystic structure and the uretero‐vesical junction, CT excretory urography (CT‐EU) was performed. CT images were acquired before and after the intravenous (iv) injection of 640 mg I/kg iodinated contrast medium (Iopamigita Insight Agents GmbH). CT‐EU was obtained acquiring contrast‐enhanced images 20 seconds, 2, 6 and 11 minutes after iv contrast medium administration. CT scans showed bilateral abnormal caudal ureteral insertion. The caudal half of the right ureter was mildly dilated, with normal dorsolateral insertion on the caudal urinary bladder wall (trigone region). Soon after its intramural course, the right ureter presented a large well‐defined oblong, space‐occupying cystic‐like dilation in the inner (mucosal/submucosal) layer of the right dorsolateral urinary bladder neck wall.

**FIG 2:**
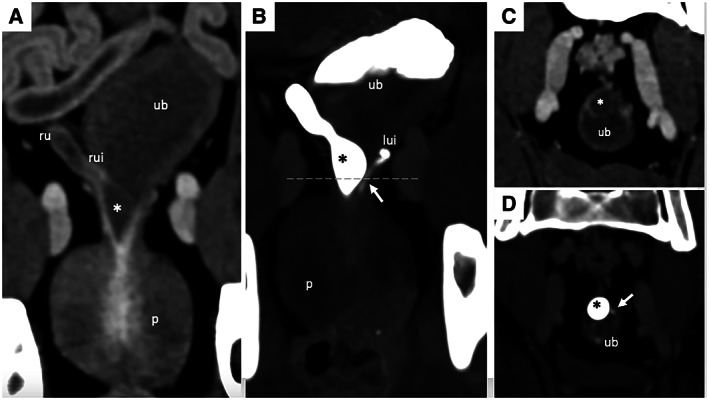
(A) Dorsal multiplanar reformatted early contrast‐enhanced CT image of the urinary bladder (ub). (B) Dorsal multiplanar reformatted maximum intensity projection delayed contrast‐enhanced (excretory phase) CT image of the urinary bladder. (C) Transverse early contrast‐enhanced and (D) delayed contrast‐enhanced (excretory phase) CT images of the urinary bladder neck obtained at the level of dashed line in image (B). The right ureter (ru) shows mild dilation before its insertion in the bladder wall (rui – right ureter insertion). After its intramural course, the right ureter presented a large mucosal/submucosal oblong, space‐occupying cystic‐like expansion in the right dorsolateral urinary bladder wall at the bladder neck (ureterocele – A, C, asterisk). The ureterocele completely filled with contrast medium, fading abruptly at the beginning of prosthatic urethra (B, D). The left ureter insertion (lui) was seen dorsolaterally on the urinary bladder neck, 7 mm caudally to the right ureter insertion. After 7 mm of intramural course (B, D – arrows), its luminal contrast enhancement faded at the level of the cranial prostatic margin. P Prostate

**FIG 3:**
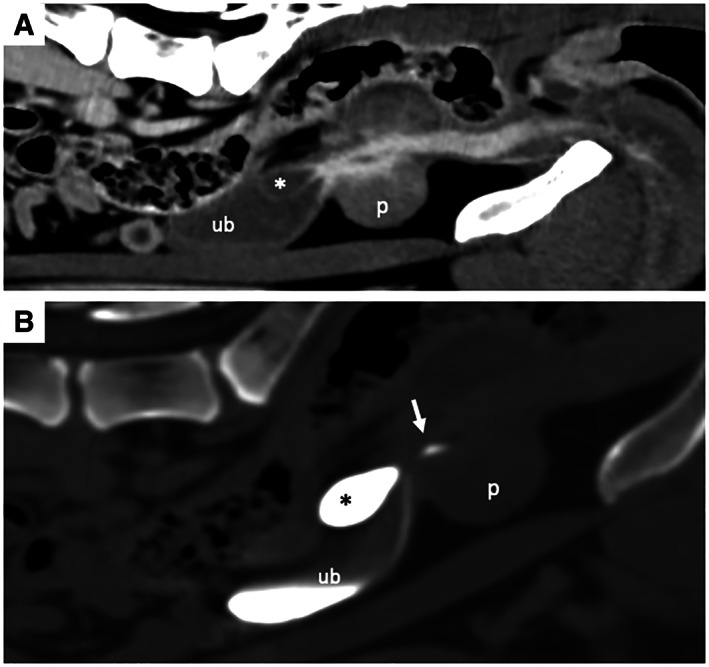
(A) Right parasagittal multiplanar reformatted early contrast‐enhanced and (B) delayed contrast‐enhanced CT images of the urinary bladder (ub). Note the right ureterocele occupying the bladder neck (*) and segmental contrast enhancement in the first 10 mm of the prostatic urethra during the most delayed excretory phase. This last finding did not rule out a bilateral or unilateral ureteral opening at the level of the cranial prostatic urethra

The intramural ureteral dilation (ureterocele) measured 15 mm in length with a transverse section of 8×8 mm, and a fluid content comparable to the bladder urine (45 Hounsfield units). In delayed excretory phases, the right ureterocele was completely filled with contrast medium, fading abruptly at the beginning of the prostatic urethra. The left ureter had a normal diameter along its entire course, with left dorsolateral insertion on the urinary bladder neck, 7 mm caudal to the right ureter insertion. After 7 mm of intramural course, the ureteral luminal contrast enhancement faded at the cranial prostatic margin (Figs 2 and 3).

The most delayed excretory phase images showed segmental contrast enhancement in the first 10 mm of the prostatic urethra (Fig 3B).

A final diagnosis of a right‐sided grade 2 ureterocele (Stiffler *et al*. 2002) and left ectopic ureter was made. A surgical approach to correct the ureteral ectopia and to resect the ureterocele was proposed to avoid a possible future onset of incontinence and prevent renal damage associated with UVJ obstruction but refused by the owner.

**FIG 4:**
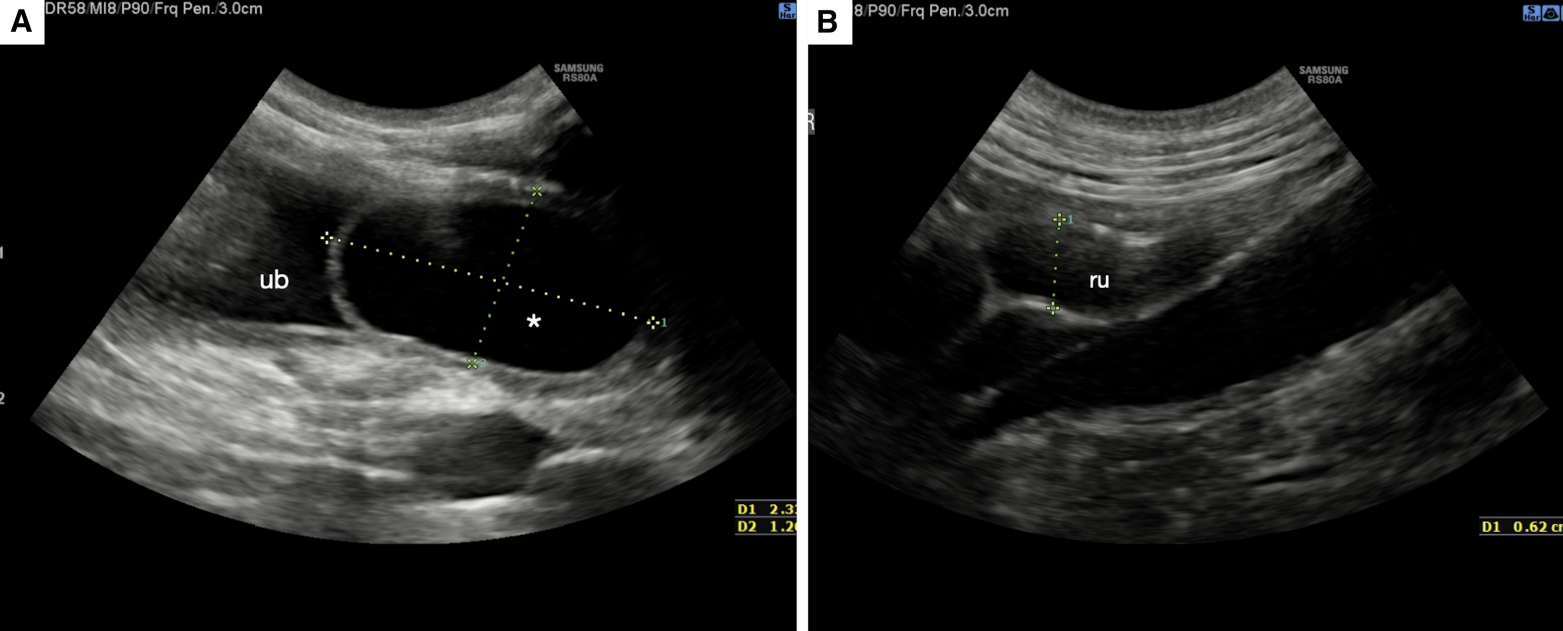
Sagittal view of the urinary bladder (ub) obtained 4 months after the initial examination (Fig 1). (A) The enlarged cystic lesion (*) measured approximately 2.3×1.2 cm. (B) A moderately enlarged distal right ureter was also observed (ru)

Four months later, the dog was presented again with pollakiuria, prolonged urination time and weak urine stream. Abdominal palpation and US of the urinary tract revealed a replete urinary bladder. At US, the right‐sided ureterocele appeared increased in size (approximately 2.3×1.2 cm) compared to the previous examination (Fig 4A). A mild right‐sided pyelectasis and moderate hydroureter (0.6 cm) were also evident (Fig 4B).

CBC and complete biochemical panel were unremarkable. A urine sample obtained by cystocentesis revealed haematuria and struvite crystals. The urine culture was negative.

**FIG 5:**
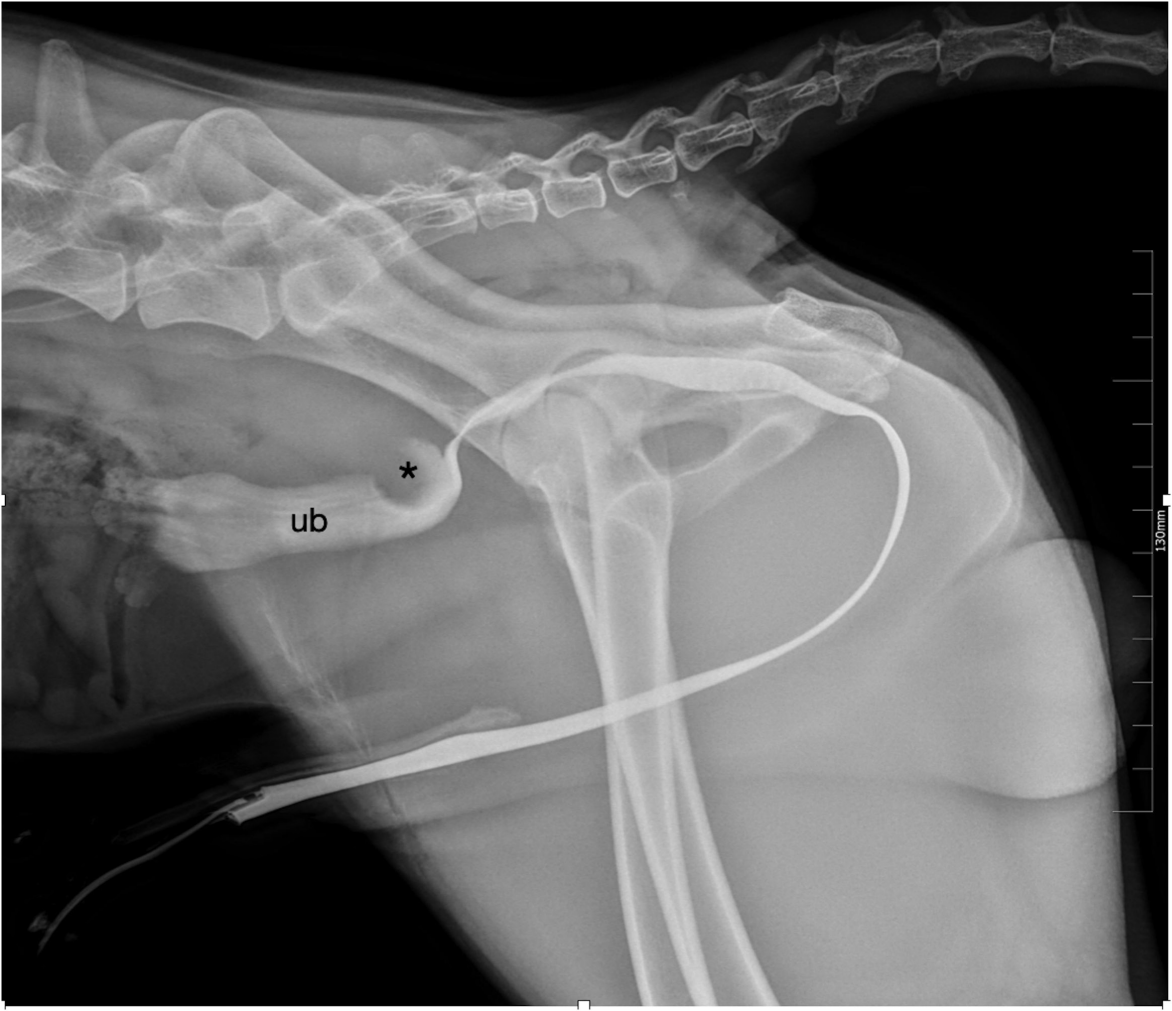
Retrograde positive contrast urethrocystography. Lateral view radiograph. The ureterocele (*) is visible as a smoothly marginated filling defect in the caudodorsal aspect of the urinary bladder neck lumen (ub)

Retrograde positive contrast urethrocystography was performed to assess the cause of pollakiuria and urinary retention. On lateral view, the urethral lumen completely filled with positive contrast medium (Iopamigta Insights Agents GmbH), ruling out urethral obstruction. However, a smoothly marginated filling defect corresponding to the previously identified ureterocele (Fig 5) was revealed. It was hypothesised that by partially occupying the caudal portion of the urinary bladder neck lumen, the ureterocele was responsible of the impaired urine flow.

A surgical approach was proposed, and the owner consented to surgical resection of the ureterocele aimed at resolving the partial obstructive condition, but refused left ureteral reimplantation. Cystotomy and ureterocele resection were therefore performed.

**FIG 6:**
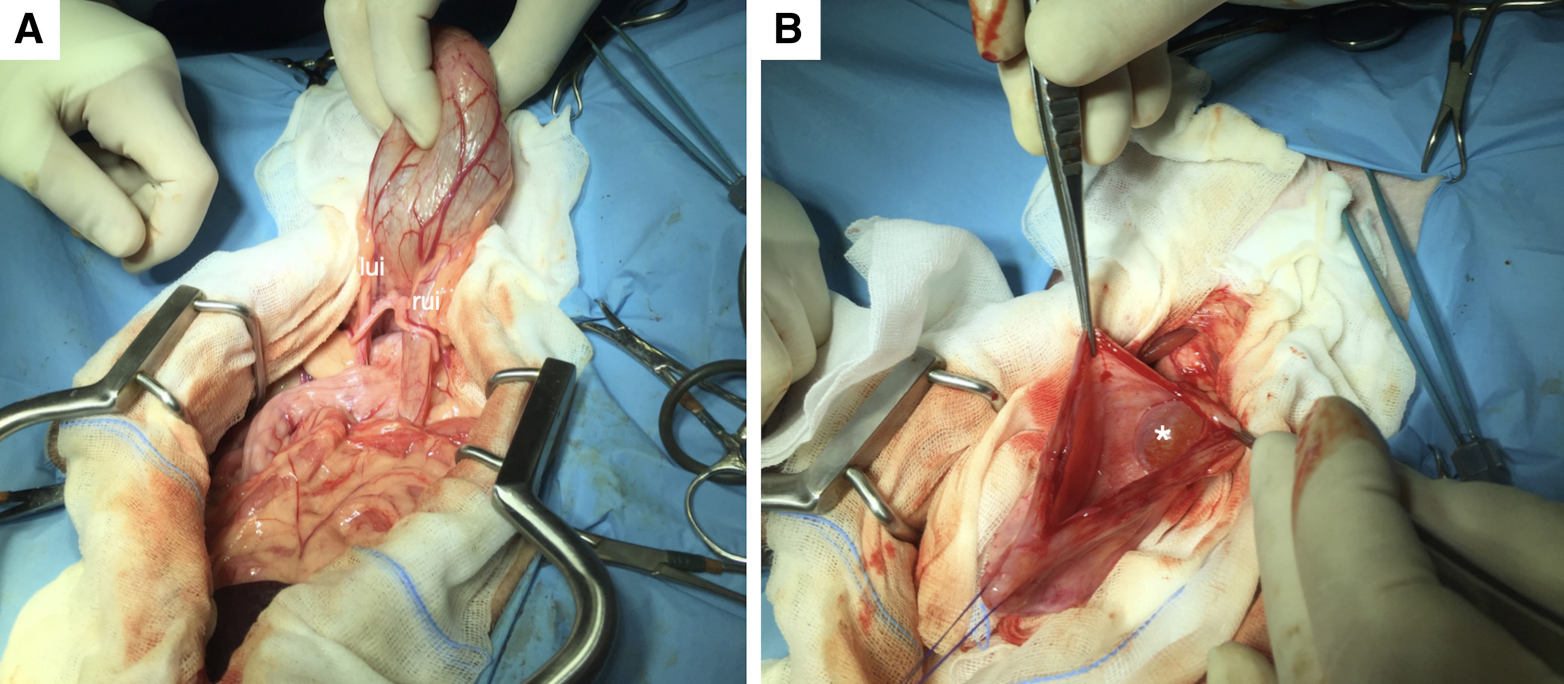
Intra‐operatory photographs. (A) Both insertions of right (rui – right ureter insertion) and left (lui – left ureter insertion) ureters were considered in a correct position. (B) Bladder mucous cystic dilation (*)

**FIG 7:**
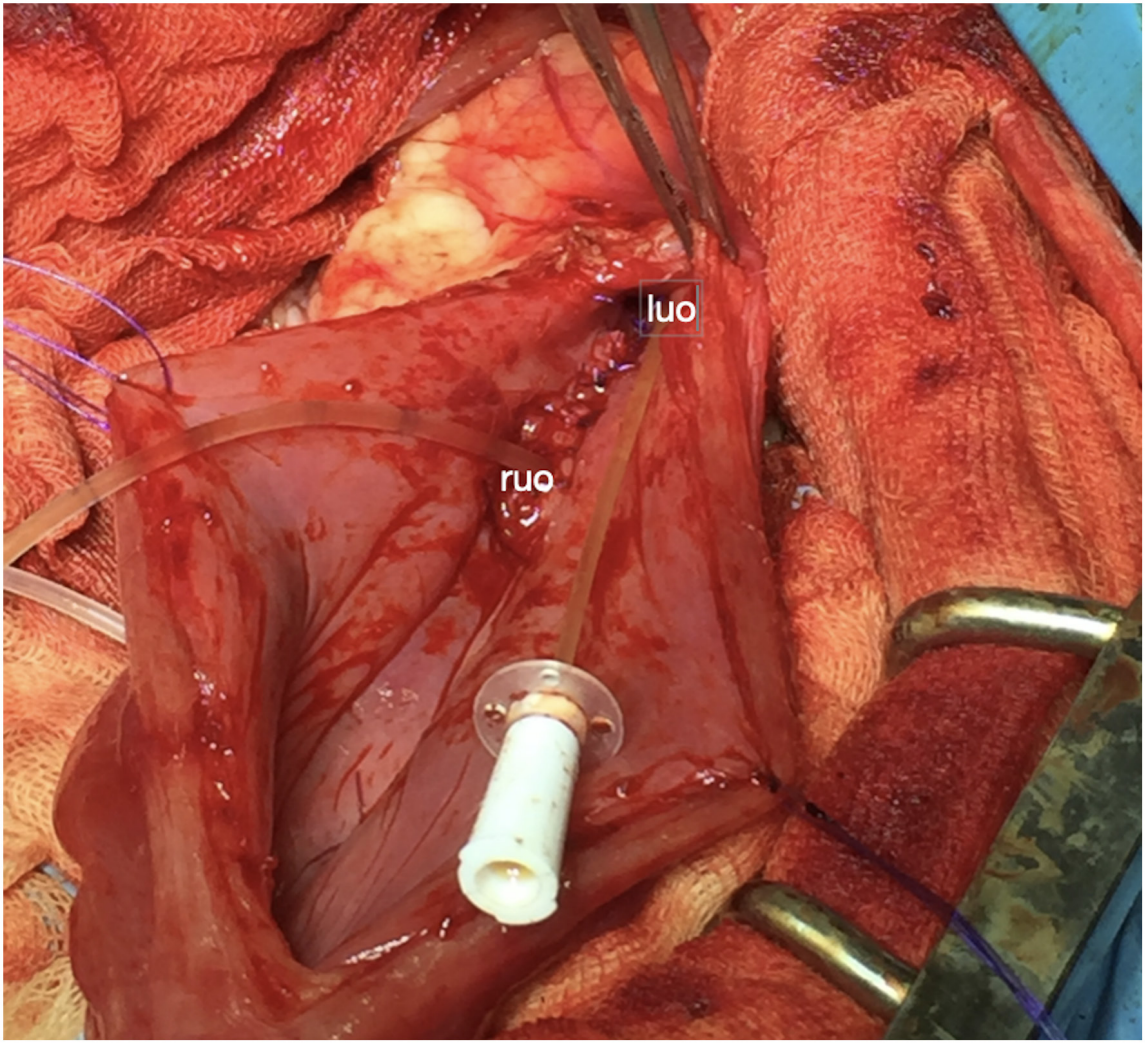
Intra‐operatory image after ureterocelectomy: a urinary catheter (Buster dog catheter 1.6 mm × 500 mm) inserted through a wide opening within the cranial pole of the cyst corresponding to the right ureteral outlet (ruo). A second catheter (Buster cat catheter 1.0 mm × 130 mm) is inserted in the left ectopic ureteral papilla (luo)

During the celiotomy, the visible urinary tract was inspected; insertions of the right and left ureter were considered correct (Fig 6A). Once the cystotomy was complete, the cystic structure was visible (Fig 6B). There appeared to be no link between the ureterocele and the bladder lumen. An incision was made on the fluid‐filled structure, and the contents of the cyst were collected for a chemical–physical examination and bacterial culture. After the ureterocelectomy, a wide opening within the cranial pole of the cyst was evident. A urinary catheter inserted through the orifice ascended the right ureter, thus identifying the opening with the right ureteral orifice (UO). The left ureteral papilla was also catheterized confirming the left ectopia (Fig 7).

**FIG 8:**
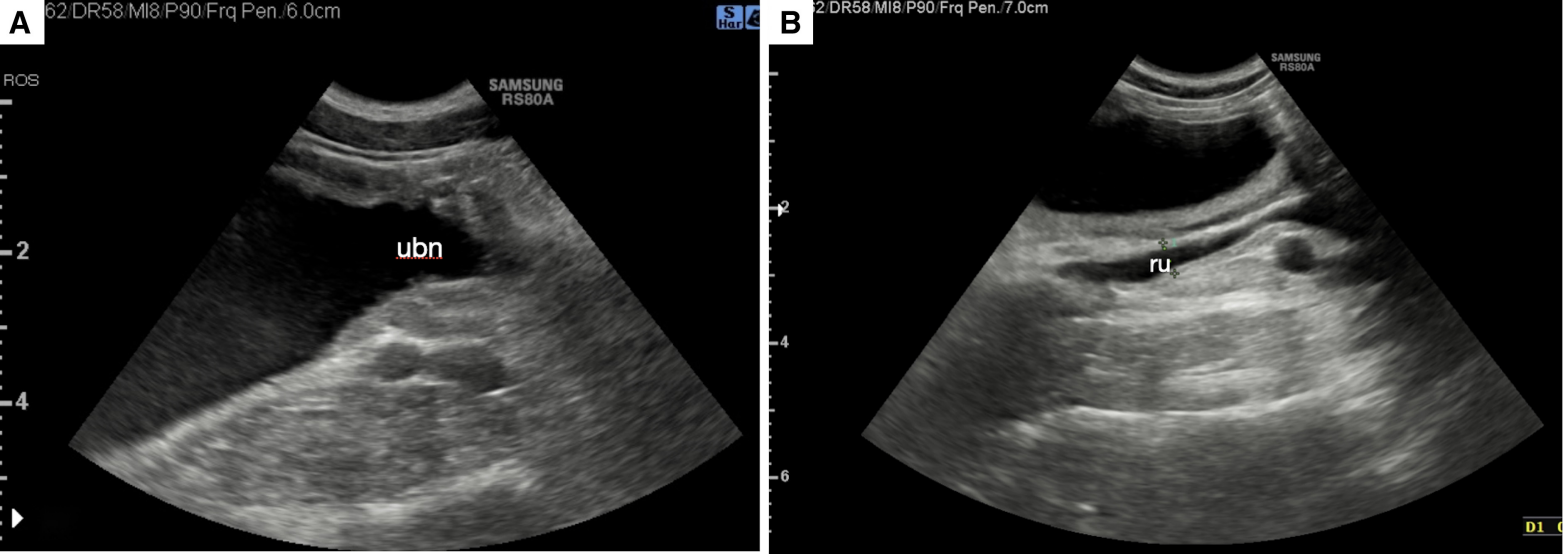
Ultrasound (US) imaging of urinary bladder and right ureter acquired 24 hours after surgical treatment. (A) Smooth fluid‐filled lesion was no longer found at the urinary bladder neck (ubn) lumen. (B) The right ureter is smaller than in the previous US images (Fig 4)

The dog was discharged 36 hours later. No bacterial growth was observed in the urine sample collected during the surgery. At the 4‐week follow‐up, the owner reported an initial phase of incontinence, which resolved spontaneously 10 days afterwards with a current normal urination behaviour. The US confirmed complete remission of pelvis dilation and hydroureter; and the ureterocele was no longer visible (Fig 8).

## DISCUSSION

This case describes, for what we believe is the first time, an acquired partial urinary obstruction caused by an intravesical ureterocele in an adult dog. The ureterocele was complicated by rapid onset urinary flow impairment and the remission of pollakiuria after surgical ureterocele resection supported the hypothesis that the enlargement of the ureterocele caused partial bladder neck obstruction.

Based on this case report and the available literature (Lee *et al*. 2020), we suggest a new simplified anatomical and functional classification system, also accounting for the presence of LUTD and urethral outflow obstruction. From an anatomical point of view, the proposed system defines an ureterocele as intravesical, if entirely within the bladder, or urethral if partly or completely situated in the urethra.

To date, only one case of the urethral localition of an ureterocele has been described in dogs (Lee *et al*. 2020). From a functional point of view, the ureterocele is classified as complicated or not complicated according to the presence or not of upper urinary tract diseases or LUTDs (Table 1).

**Table 1:** Proposed anatomical and functional classification system

Classification system	
Anatomical	Functional
Intravesical	Not complicated: no evidence of upper or lower urinary tract disease
	Complicated: evidence of upper or lower urinary tract disease Presence of upper or lower urinary tract disease related to ureterovesical obstruction (*i.e*. hydroureter and/or obstructive nephropathy) or incomplete bladder emptying (*i.e*. recurrent cystitis, UTIs, crystalluria and/or bladder stones) and/or impairment of urine flow
Urethral	Not complicated: no evidence of upper or lower urinary tract disease
	Complicated: evidence of upper or lower urinary tract disease Presence of upper or lower urinary tract disease related to ureterovesical obstruction (*i.e*. hydroureter and/or obstructive nephropathy) or incomplete bladder emptying (*i.e*. recurrent cystitis, UTIs, crystalluria and/or bladder stones). Urethral obstruction with acute or chronic difficult urination

UTI Urinary tract infection

In dogs, ureteroceles are associated with ectopic ureter in 60% of cases (Pearson & Gibbs 1971, Smith & Park 1974, Stowater & Springer 1979, Ross & Lamb 1990, Lamb & Gregory 1998, Lautzenhiser & Bjorling 2002, Tattersall & Welsh 2006, Colopy *et al*. 2011, Green *et al*. 2011, Lorigados *et al*. 2012, Heier *et al*. 2019, Rogatko *et al*. 2019, Anderson *et al*. 2020) and are reported as bilateral in approximately 13% of cases (Pearson & Gibbs 1971, Smith & Park 1974, Hoffman *et al*. 1991, Secrest *et al*. 2011, Rogatko *et al*. 2019). In our case, during surgery, the UO remained unidentified as reported in 50% of cases in a previous case series in dogs (Rogatko *et al*. 2019).

Despite the presence of a left ectopic ureter, the dog showed no incontinence either before or after the removal of the ureterocele. It has been suggested that the longer urethra of males is better able to counter the distal flow of urine, which allows retrograde filling of the bladder (McLoughlin & Chew 2000).

Both ureterocele and ureteral ectopia are associated with a major risk of UTI (Sutherland‐Smith *et al*. 2004). In this dog, UTI was excluded by bacterial culture. However, previous UTIs cannot be excluded, and the bacterial culture result may have been affected by previous empirical antibiotic treatments.

Obstructive urinary retention is common in small animals and should be carefully evaluated because it increases the risk of infection and can induce various complications. This case report shows that, although ureterocele is a rare condition in dogs, it should be considered as a differential diagnosis in patients with urinary obstruction and pollakiuria.

## Conflict of interest

None of the authors of this article has a financial or personal relationship with other people or organisations that could inappropriately influence or bias the content of the paper.
